# Model to improve specificity for identification of clinically-relevant expanded T cells in peripheral blood

**DOI:** 10.1371/journal.pone.0213684

**Published:** 2019-03-14

**Authors:** Julie Rytlewski, Shibing Deng, Tao Xie, Craig Davis, Harlan Robins, Erik Yusko, Jadwiga Bienkowska

**Affiliations:** 1 Adaptive Biotechnologies, Seattle, Washington, United States of America; 2 Pfizer Incorporated, La Jolla, California, United States of America; 3 Fred Hutchinson Cancer Research Center, Seattle, Washington, United States of America; Peter MacCallum Cancer Centre, AUSTRALIA

## Abstract

Current methods to quantify T-cell clonal expansion only account for variance due to random sampling from a highly diverse repertoire space. We propose a beta-binomial model to incorporate time-dependent variance into the assessment of differentially abundant T-cell clones, identified by unique T Cell Receptor (TCR) β-chain rearrangements, and show that this model improves specificity for detecting clinically relevant clonal expansion. Using blood samples from ten healthy donors, we modeled the variance of T-cell clones within each subject over time and calibrated the dispersion parameters of the beta distribution to fit this variance. As a validation, we compared pre- versus post-treatment blood samples from urothelial cancer patients treated with atezolizumab, where clonal expansion (quantified by the earlier binomial model) was previously reported to correlate with benefit. The beta-binomial model significantly reduced the false-positive rate for detecting differentially abundant clones over time compared to the earlier binomial method. In the urothelial cancer cohort, the beta-binomial model enriched for tumor infiltrating lymphocytes among the clones detected as expanding in the peripheral blood in response to therapy compared to the binomial model and improved the overall correlation with clinical benefit. Incorporating time-dependent variance into the statistical framework for measuring differentially abundant T-cell clones improves the model's specificity for T-cells that correlate more strongly with the disease and treatment setting of-interest. Reducing background-level clonal expansion, therefore, improves the quality of clonal expansion as a biomarker for assessing the T cell immune response and correlations with clinical measures.

## Introduction

High-throughput next-generation sequencing of the T cell receptor (TCR) repertoire, i.e., immunosequencing, enables precise molecular identification and tracking of tens to hundreds of thousands of T-cell clones in a single subject [1]. A key component of the adaptive immune system is the clonal expansion of activated T cells. With immunosequencing, clonally expanded T cells can be identified by comparing the frequency of each T-cell clone at one time point versus another. One challenge with immunosequencing data is developing a systematic framework to determine if the increase in T-cell clone frequency meets the criteria for clonal expansion. Here, we describe a statistical framework that accounts for sampling and time-dependent repertoire variability to detect T-cell clones that are differentially abundant in an unbiased and quantitative manner.

In previous work by DeWitt *et al*, detection of clonally expanded T-cell clones was shown to correlate with an immune response to the yellow fever vaccine [2]. This earlier method was based on a Fisher’s exact test, which can also be implemented as a binomial test comparing two proportions. Although the binomial model only accounts for random sampling variance around clone frequency, clonal expansion detected using this method was still found to correlate with pharmacodynamic activity and clinical response across a wide range of studies in the immuno-oncology setting [3–7]. Given that the T-cell repertoire is a highly dynamic system that evolves over time, we hypothesized that incorporating time-dependent variability into the differential abundance assessment would improve specificity for clinically relevant clonal expansion by reducing the identification of T-cell clones whose frequencies are changing within the range of normal physiology.

The approach presented here uses a beta-binomial model to incorporate time-dependent variance in addition to the previously-captured sampling variance. We measured the variance in T-cell repertoires between technical replicates as well as blood samples drawn 2 and 4 weeks apart from ten healthy subjects. Variance between technical replicates did not contribute to the identification of differentially abundant clones, but time-dependent variance indeed affected this measure, resulting in tens of clones identified. We used the measured repertoire variance over a two-week interval from these healthy donors to calibrate the allowable range of time-dependent dispersion for the beta-binomial model. We then applied the calibrated beta-binomial model to a urothelial cancer cohort to measure T-cell clonal expansion in the blood after administration of an anti-PD-L1 immunotherapy. In these patients, we found that T-cell clones identified as expanded in blood by the beta-binomial model were more likely to also reside in the tumor microenvironment than clones identified with the binomial model. This enrichment for tumor infiltrating lymphocytes (TILs) among expanded T-cell clones in the peripheral repertoire also improved the overall correlation with clinical benefit.

## Results

### Comparison of the binomial model in technical replicates and time-course blood samples

**Fig 1:**
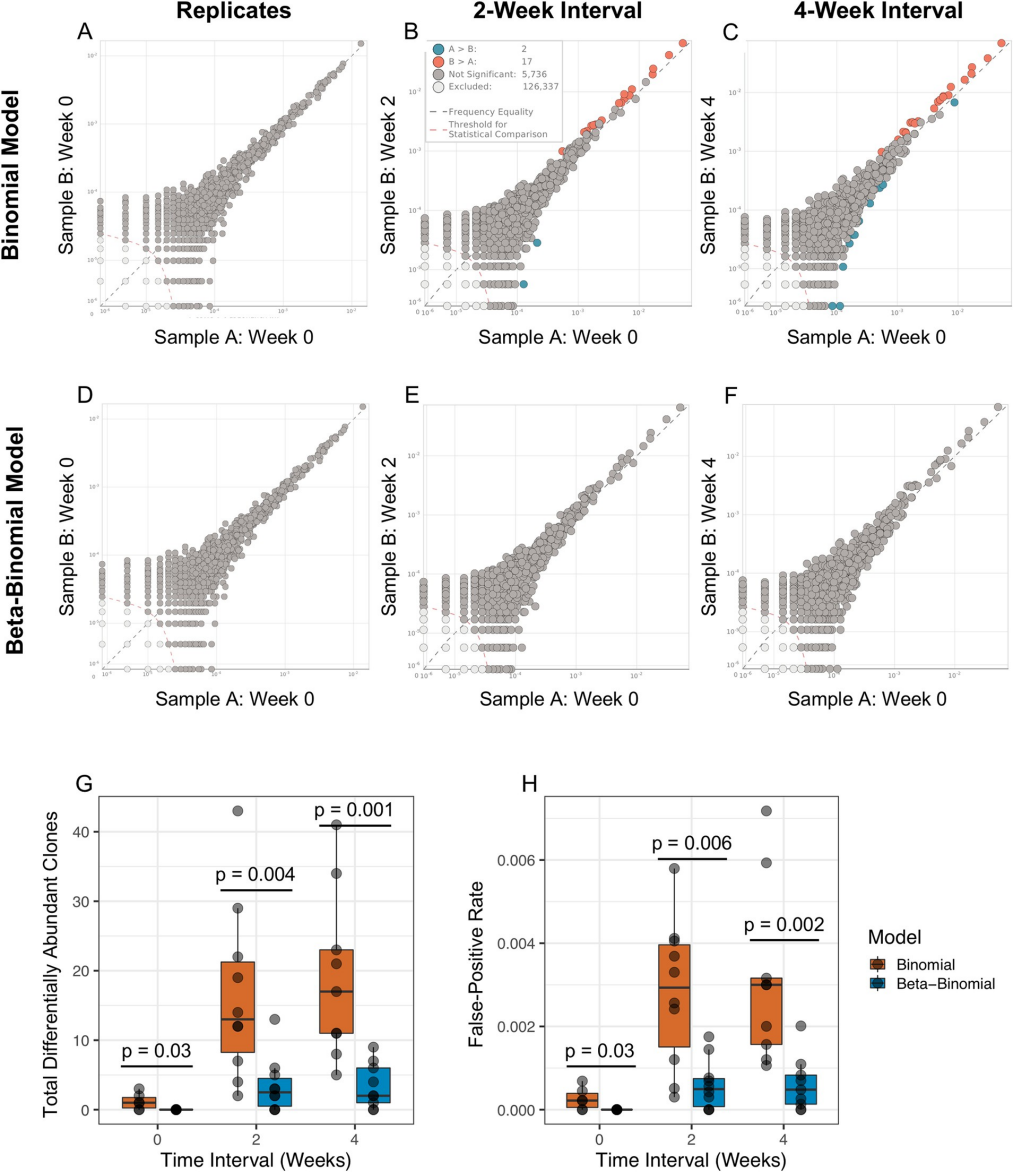
Differential abundance analysis in technical replicates and samples collected over 2-week and 4-week intervals. Scatter plots of T-cell clone frequencies (from the whole blood of a single donor) in technical replicates as well as samples collected every two weeks over a 4-week period, with differentially abundant clones annotated in orange or cyan as determined by the binomial model (A–C) or the beta-binomial model (D–F). For each time interval, T-cell clones with were tested for differential abundance (dark grey, orange, and cyan) and the remaining clones (light grey) were excluded. Summary of the differential abundance analysis results comparing the performance of the binomial and beta-binomial model in detecting differentially abundant clones (G) and the overall false-positive rate (H). Statistical significance was assessed in G and H by Wilcoxon Rank Sum tests.

To characterize the performance of the binomial model (Eq 1) and illustrate the importance of accounting for time-dependent biological variance, we analyzed T-cell clone frequencies in technical replicates as well as samples collected at 2-week and 4-week intervals. Fig 1A shows clone frequencies in a pair of technical replicates, which were sequenced on two aliquots from the same gDNA pool. Application of the binomial model returned an average of 1.2 differentially abundant clones across six comparisons performed between four technical replicates, resulting in a false-positive-rate of 2.6E-4 (Fig 1A). In contrast, an average of 16.4 differentially abundant clones at a false-positive-rate of 0.0028 were identified over a 2-week interval (Fig 1B) and an average of 19 differentially abundant clones at a false-positive-rate of 0.0031 were identified over a 4-week interval (Fig 1C). Furthermore, the false discovery rate was higher for clones above 0.1% using the binomial model due to increased power for detecting small changes in clone frequency.

**Fig 2:**
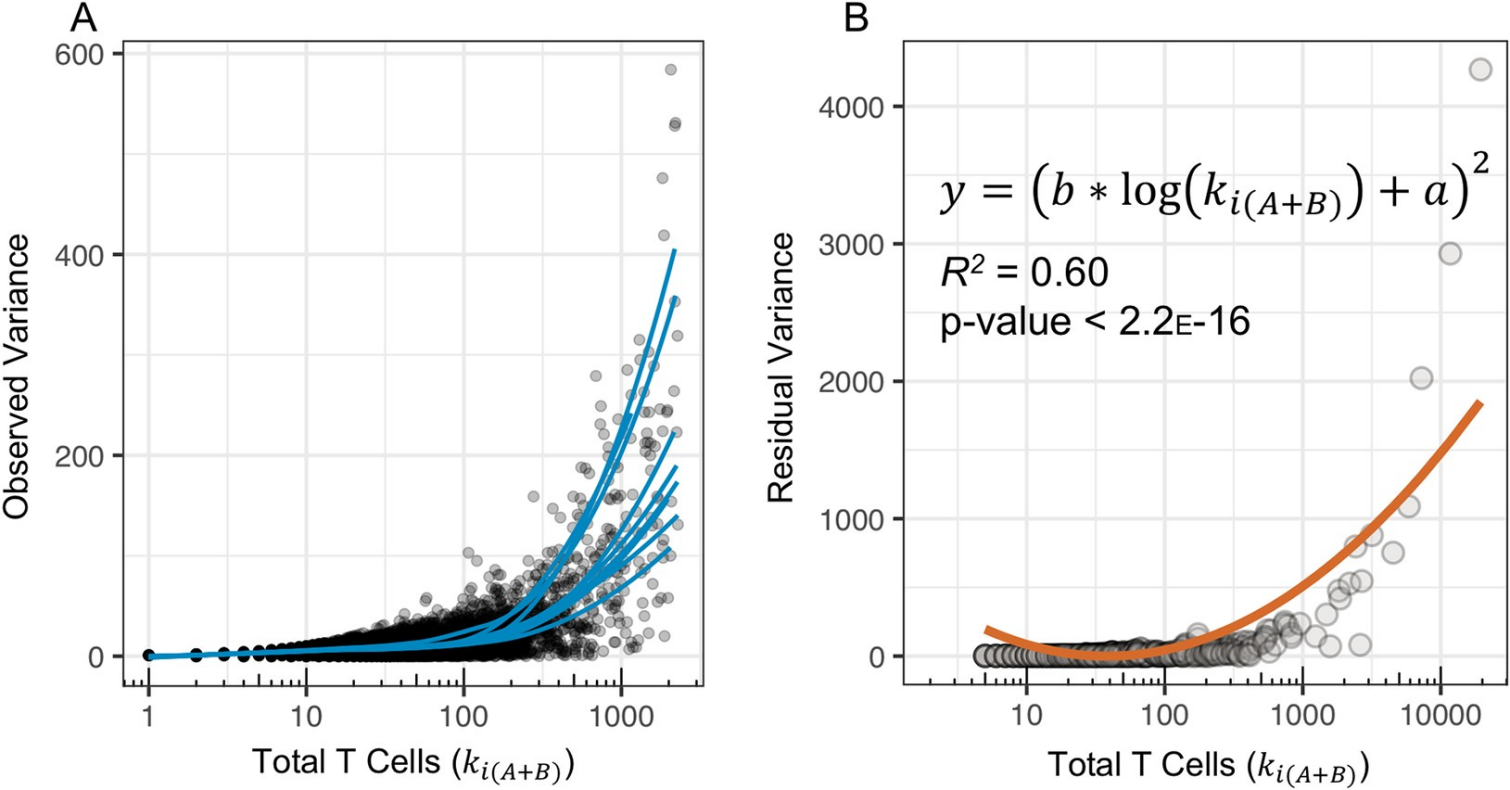
Dispersion parameter as a function of clone frequency in 10 healthy donors at week 2 and at week 4. (A) The observed variance of |*k*_*iA*_−*k*_*iB*_| plotted for each n_t_ across all ten subjects; blue lines are LOESS curves representing the data trends of each subject. (B) Fit of the modeled residual variance (orange line), defined as (b*log(*k*_*i*(*A*+*B*)_)+*a*)^2^, to the observed residual variance (gray points) in T-cell counts between two samples from one blood donor; observed residual variance was calculated as the absolute difference between the observed variance of |*k*_*iA*_−*k*_*iB*_| and the expected variance due to binomial sampling, which follows the form *np*(1−*p*), where *n* is equal to *k*_*iA*_ and *p* is the frequency of observing a given pair of *k*_*iA*_ and *k*_*iB*_ for each value of *k*_*i*(*A*+*B*)_.

In order to account for this time-dependent variability in T-cell clone frequency, we first characterized the observed variance in T cell counts between a given clone over a two-week interval across all 10 subjects, defined as |*k*_*iA*_−*k*_*iB*_|. A linear mixed-effects model on log_10_-transformed data found that the variance in T-cell counts across all subjects significantly increased with the total T cell count, *k*_*i*(*A*+*B*)_ (p < 0.001; Fig 2A). The residual variance, calculated by subtracting the expected binomial variance from the observed variance, accordingly increased for larger values of *k*_*i*(*A*+*B*)_ and fit the modeled form, *v* = (b*log(*k*_*i*(*A*+*B*)_)+*a*)^2^ (*R*^*2*^ = 0.60, p << 0.001; Fig 2B). This approach permitted clone frequency and total T-cell count to define the beta probability density and ultimately the additional variance incorporated by the beta-binomial model in Eq 2. Consequently, normal biological time-dependent variance in T-cell clone frequencies could be accounted for when assessing statistical changes in clone frequency between two samples (Fig 1D–1F). Compared to the previous results with binomial model, we found that the beta-binomial model significantly reduced the number of clones detected as differentially abundant as well as the subsequent false-positive rate (Wilcoxon Rank Sum Test, p < 0.05) across all time intervals (Fig 1G and 1H).

### Characterization of statistical power

**Fig 3:**
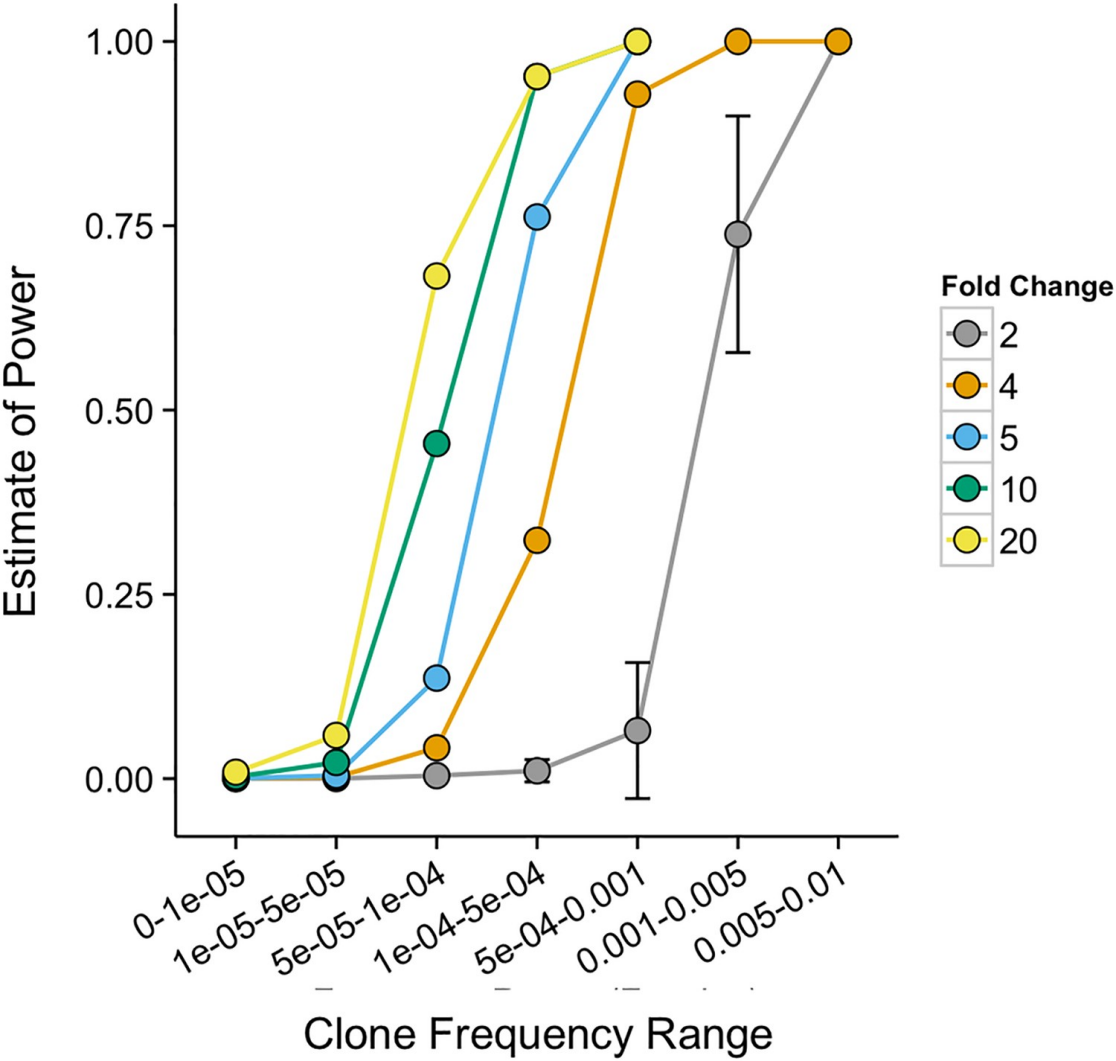
Estimated statistical power to detect significantly expanded clones as a function of pre-treatment clone frequency. Comparing T-cell repertoires created from mixtures of samples enabled identification of T-cell clones with expected changes in frequencies relative to a baseline sample. Power, defined as the probability of detecting a clone as significantly greater in frequency in sample B, is plotted versus baseline frequency (Sample A, x-axis) and the expected fold change based on the mixtures being compared. The dashed line indicates the frequency bin at which the model requires a minimum total template count of 5 between sample A and B, which reduces the number of T-cell clone frequencies being tested for significance.

Statistical power to identify differentially abundant clones was estimated as a function of initial clone frequency and frequency fold-change using a dilution experiment in which repertoires from two different individuals were mixed at a range of specified ratios. These fixed ratios allow us to control the expected fold-change in clone frequency when comparing two different mixtures. Fig 3 shows the estimate of statistical power versus clone frequency and frequency fold-change, ranging from 2-fold up to 20-fold. As expected, we have greater statistical power to identify a clone as differentially abundant if its initial frequency is higher and/or the fold-change in its frequency between the two samples is greater.

### Evidence for enrichment of TIL clones

**Fig 4:**
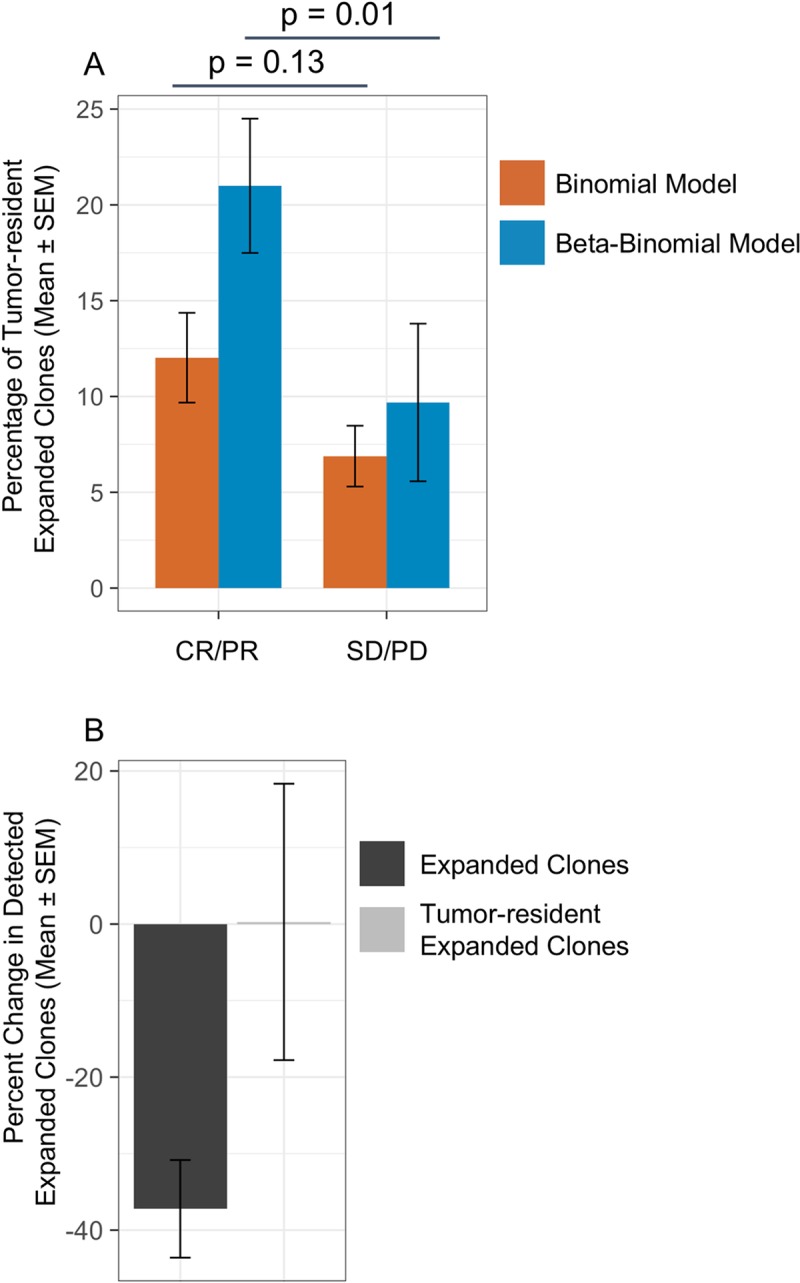
Comparison of the binomial and beta-binomial models of differential abundance and their ability to enrich for expanded TILs in the peripheral repertoire of responders to IO treatment. (A) Percentage of expanded clones in the peripheral repertoire also found in the pre-treatment tumor repertoire, i.e., TILs, for a cohort of urothelial patients treated with atezolizumab (n = 26). Patients were categorized by RECIST outcomes as responders (CR/PR) or non-responders (SD/PD). (B) Percent change in the number of peripheral repertoire clones identified as expanded and the subset of circulating TIL clones expanded with the beta-binomial model relative to the binomial model. Error bars represent standard error of the mean.

We re-analyzed a previously published urothelial cancer cohort from Snyder *et al* (Complete/Partial Responders: n = 14; Stable/Progressive Disease: n = 22) to compare the detection of TILs expanded in the peripheral repertoire with the beta-binomial and binomial models [3]. T-cell clones identified as expanded in the blood by each model were annotated as TILs based on their presence in pre-treatment tumor samples. As shown in Fig 4A, the beta-binomial not only increases the statistical resolution between responders (CR/PR) and non-responders (SD/PD) over the binomial model (p = 0.01 vs p = 0.13) but also increases the proportion of expanded clones identified as TILs for patients responding to therapy (p = 0.08). The enhanced specificity for the peripheral expansion of TILs with the beta-binomial is largely due to fewer clones being identified as expanded rather than an increase in TIL clones, with mean differences of approximately -40% and 0% relative to the binomial model, respectively (Fig 4B).

## Discussion

In healthy individuals, we found that the number of T-cell clones detected as expanded increases as the time interval between Sample A and B increases. This time-dependence highlights the importance of establishing a prior on typical biological variance within the T-cell repertoire, especially over weekly and monthly time intervals commonly used for collecting clinical trial patient samples. We expect that the biological variance may further increase if much larger time intervals were used, e.g., months to years. By training a new beta-binomial model with data from healthy individuals, identification of differentially abundant T-cell clones now accounts for both random sampling from a diverse repertoire and normal time-dependent biological variability. In effect, the clonal expansion measured by the beta-binomial differential abundance model should display reduced biological noise and subsequently enrich for clones responding to the pharmacologic or pathologic setting-of-interest.

In a cohort of urothelial cancer patients treated with atezolizumab, expansion of TILs in the peripheral repertoire was previously reported to correlate with RECIST assessment [3]. Other studies have similarly analyzed the overlap between TILs and the peripheral repertoire and found correlations between patient outcomes or drug activity and expansion of T-cell clones [6,8–10]. The association of peripherally expanded TILs with treatment outcomes has identified them as putative therapeutic effectors and as valuable biomarkers of clinical efficacy. The re-analysis presented here of the urothelial cancer cohort demonstrated that the beta-binomial model, by accounting for biological variability over time, reduces the total number of clones identified as expanding without affecting the number of TIL clones detected. Consequently, this model enriches for TIL clones within the expanded T-cell population in the blood compared to the binomial model and strengthens the association between expanded clones and clinical benefit. This strengthened association with clinical outcomes suggests that the statistical framework presented here a) represents an improvement over existing methods to quantify clonal expansion and b) may be similarly applicable as a biomarker for assessing pharmacodynamic activity and patient response to therapy.

However, tumor samples are not always available for sequencing. We, and others, have also found that assessment of clonal expansion from longitudinal blood samples alone can be leveraged for optimizing therapeutic dosing and timing regimens as well as the assessment of novel drug combinations [5,7,11]. In addition, companion diagnostics are now increasingly common to identify patients for which there is an *a priori* expectation of benefit. Hence, early measures or predictors of therapeutic response, such as clonal expansion, have broad clinical value.

## Methods

### Training cohort

Whole blood samples collected from 10 healthy donors at three time points with 2-week intervals between collections were purchased from AllCells, LLC (Emeryville, CA). Among the 10 donors, there were 3 females and 7 males with ages ranging from 29 to 64 with an average age of 43. No immune events such as infections were documented for these donors over the course of collection. Genomic DNA was extracted at Adaptive Biotechnologies (Seattle, WA) for subsequent immunosequencing.

### Immunosequencing

Immunosequencing of the CDR3 regions of human TCRβ chains was performed using the immunoSEQ Assay (Adaptive Biotechnologies, Seattle, WA). Extracted genomic DNA was amplified in a bias-controlled multiplex PCR, followed by high-throughput sequencing. Sequences were collapsed and filtered in order to identify and quantitate the absolute abundance of each unique TCRβ CDR3 region for further analysis as previously described [1].

### Statistical method for identification of expanded T cell clones in blood

DeWitt *et al* previously reported a method for identifying differentially abundant T-cell clones between two samples [2]. The method employed Fisher’s exact test to compute a p-value for each T-cell clone, identified by a unique TCRβ rearrangement, against the null hypothesis that the T-cell clone frequency was the same in both Sample A and Sample B. In practice, this procedure can also be implemented in terms of the binomial distribution to estimate the probability (*θ*_*i*_) for each T-cell clone *i* of counting *k*_*i*_ templates given *n* total T cells in a sample according to Eq 1:

$$P(k_{i}|\theta_{i},n)=\frac{n!}{k_{i}!(n-k_{i})!}\theta_{i}^{k_{i}}{\left(1-\theta_{i}\right)}^{n-k_{i}}$$ (1)

Eq 1 permits testing against the null hypothesis that *θ*_*A*_ = *θ*_*B*_ = *θ*, where *θ* can be estimated from (*k*_*iA*_+*k*_*iB*_)/(*n*_*A*_+*n*_*B*_), where subscript *A* denotes Sample A and *B* denotes Sample B; in other words, this binomial implementation is testing the null hypothesis that the frequency in Sample B, compared to the average frequency between the two samples, is within the binomial variance expected from sampling *n* T cells in Sample B.

In Eq 1, variance in observed clone frequencies is expected to arise from sampling a diverse pool of T cells and driven solely by actual clone frequency and the number of T cells analyzed. To incorporate additional variance into the model with the goal of modeling natural, time-dependent variation of T-cell clone frequencies, we incorporated the beta distribution as a prior for the binomial parameter *θ* in a beta-binomial model. The beta distribution yields a probability density function for each clone frequency, parameterized by two shape parameters β_1_ and β_2_. Thus, incorporating the beta probability density into Eq 1 permits additional variance around the clone frequency *θ* and estimates the probability of observing a given frequency in Eq 2 [12,13]:

$$P(\theta |k_{i},n,\beta_{1},\beta_{2})\propto \theta^{k_{i}+\beta_{1}-1}{\left(1-\theta \right)}^{n-k_{i}+\beta_{2}-1}$$ (2)

In implementing the posterior distribution of Eq 2, we reparametrized the shape coefficients with mean frequency, *μ*, and variance, *v*, by *β*_1_ = *μv* and *β*_2_ = (1−*μ*)*v* [12]. Using this reparametrized implementation, we trained and modeled coefficients *β*_1_ and *β*_2_ as a function of the total template counts observed for a T-cell clone in Sample A and Sample B. The training method involves determining the mean frequency of all observations at given total template count, *k*_*i*(*A*+*B*)_ = *k*_*iA*_+*k*_*iB*_, and modeling variance as *v* = (b*log(*k*_*i*(*A*+*B*)_)+*a*)^2^, where *k*_*iA*_ is the template count in sample A and *k*_*iB*_ is the templates count in sample B. To apply the implementation of Eq 2 after training, the mean frequency is estimated from *k*_*i*(*A*+*B*)_/*n*_(*A*+*B*)_ and the variance is determined from the modeled variance with total template counts in Sample A and B, *k*_*iA*_+*k*_*iB*_, as input parameters to determine *β*_1_ and *β*_2_ for that clone frequency. The python script implementing both the binomial and beta-binomial models and associated data is available at: https://github.com/jrytlewski/beta_binomial_paper.

To determine p-values, we calculate and sum the exponent of the log-likelihood of the beta-binomial model (Eq 1 with the Eq 2 posterior) for each template count ranging from 0 to *k*_*iA*_ for a one-sided test. For a two-sided test, we repeat the summation for all template counts yielding a point estimate probability value greater than and less than the count observed. To control for multiple testing, we excluded T-cell clones where *k*_*i*(*A*+*B*)_<5 and employ the Benjamini-Hochberg (BH) correction. We considered T-cell clones with a BH-adjusted false discovery rate (FDR) less than 0.01 significant and differentially abundant and identified these rearrangements as expanded or contracted clones based on the direction of their frequency change.

## References

[1] Carlson CS, Emerson RO, Sherwood AM, Desmarais C, Chung M-W, Parsons JM, et al Using synthetic templates to design an unbiased multiplex PCR assay. Nat Commun. Nature Publishing Group; 2013;4: 1–9. 10.1038/ncomms3680 24157944

[2] DeWitt WS, Emerson RO, Lindau P, Vignali M, Snyder TM, Desmarais C, et al Dynamics of the cytotoxic T cell response to a model of acute viral infection. J Virol. 2015;89: 4517–4526. 10.1128/JVI.03474-14 25653453 PMC4442358

[3] Snyder A, Nathanson T, Funt SA, Ahuja A, Buros Novik J, Hellmann MD, et al Contribution of systemic and somatic factors to clinical response and resistance to PD-L1 blockade in urothelial cancer: An exploratory multi-omic analysis. Minna JD, editor. PLoS Med. 2017;14: e1002309–24. 10.1371/journal.pmed.1002309 28552987 PMC5446110

[4] Roh W, Chen P-L, Reuben A, Spencer CN, Prieto PA, Miller JP, et al Integrated molecular analysis of tumor biopsies on sequential CTLA-4 and PD-1 blockade reveals markers of response and resistance. Sci Transl Med. 2017;9: eaah3560. 10.1126/scitranslmed.aah3560 28251903 PMC5819607

[5] Davis CB, Deng S, Xie T, Bienkowska J, DeWitt W, Rytlewski J, et al Use of TCR diversification in peripheral blood as a pharmacodynamic biomarker for immunomodulatory agents in oncology. Keystone. Montreal; 2018 pp. 1–1.

[6] Sveinbjornsson B, Camilio KA, Wang M-Y, Nestvold J, Rekdal O. LTX-315: A first-in-class oncolytic peptide that reshapes the tumor microenvironment. CIMT. 2017 pp. 1–1. 10.4155/fmc-2017-0088 28490192

[7] Hecht JR, Falchook GS, Patel M, Infante JR, Naing A, Wong DJ, et al Efficacy, Safety and Immune Activation with PEGylated Human IL-10 (AM0010) plus FOLFOX in Metastatic Pancreatic Adenocarcinoma (PDAC). ESMO. 2017 pp. 1–1.

[8] Hsu MS, Sedighim S, Wang T, Antonios JP, Everson RG, Tucker AM, et al TCR Sequencing Can Identify and Track Glioma-Infiltrating T Cells after DC Vaccination. Cancer Immunology Research. 2016;4: 412–418. 10.1158/2326-6066.CIR-15-0240 26968205 PMC4873445

[9] Forde PM, Chaft JE, Smith KN, Anagnostou V, Cottrell TR, Hellmann MD, et al Neoadjuvant PD-1 Blockade in Resectable Lung Cancer. N Engl J Med. 2018;: NEJMoa1716078. 10.1056/NEJMoa1716078 29658848 PMC6223617

[10] Page DB, Yuan J, Redmond D, Wen YH, Durack JC, Emerson R, et al Deep Sequencing of T-cell Receptor DNA as a Biomarker of Clonally Expanded TILs in Breast Cancer after Immunotherapy. Cancer Immunology Research. 2016;4: 835–844. 10.1158/2326-6066.CIR-16-0013 27587469 PMC5064839

[11] Hopkins AC, Yarchoan M, Durham JN, Yusko EC, Rytlewski JA, Robins HS, et al T cell receptor repertoire features associated with survival in immunotherapy-treated pancreatic ductal adenocarcinoma. JCI Insight. 2018;3 10.1172/jci.insight.122092 29997287 PMC6124515

[12] Navarro D, Perfors A. An introduction to the Beta-Binomial model. 2010 pp. 1–9.

[13] Kruschke JK. Doing Bayesian Data Analysis: A Tutorial with R and BUGS Academic Press Google Scholar 2011.

